# “I think office environments aren’t really conducive to physical activity”: a qualitative interview study with participants of a workplace physical activity programme

**DOI:** 10.1186/s12889-025-25007-x

**Published:** 2025-11-06

**Authors:** Samuel J. Warne, James A. Ainge, Gozde Ozakinci

**Affiliations:** 1 Population and Behavioural Science Division, School of Medicine, University of St Andrews, St Andrews, UK; 2 School of Psychology & Neuroscience, University of St Andrews, St Andrews, UK; 3 Division of Psychology, Faculty of Natural Sciences, University of Stirling, Stirling, FK9 4LA UK

**Keywords:** Walking, Exercise, Steps, Employee, Health, Fitness, Competition, Teams, COM-B model

## Abstract

**Background:**

There is a wealth of evidence surrounding the positive impacts of exercise on health and wellbeing, however, sedentary behaviour is still prevalent, particularly in the workplace. This has led to the development and popularity of workplace programmes intended to increase activity levels. An example is Step Count Challenge (SCC) available to all workplaces nationwide across Scotland. In SCC, teams of five colleagues attempt to complete as many steps as possible during either a four- or eight-week period in Autumn and Spring. The purpose of this qualitative study was to explore SCC participants’ experiences and the impact of participation on exercise behaviours, using the COM-B model.

**Methods:**

Previous SCC participants (*n* = 15) took part in one-to-one semi-structured interviews via Microsoft Teams or phone call, with conversation particularly centred around activity behaviours, both during the SCC and beyond the challenge. The interview schedule was structured such that each element of the COM-B model was addressed, and responses were analysed using a hybrid deductive-inductive analytic approach to reflexive thematic analysis.

**Results:**

Five salient themes (and subthemes) were generated to represent the data, each mapped to an element of the COM-B Model. These overarching themes were: (1) *Beliefs about capability impacted by experiences and performance*,* both personal and those of others*, (2) *SCC presents the opportunity for a Physical Activity behaviour change*, (3) *General physical activity barriers*, (4) *SCC impacting Physical Activity Motivation*, and (5) *Why do we do it?*

**Conclusions:**

Participants believed that they benefitted from the SCC, and that their physical activity levels were positively impacted as a result of their participation. This positive impact seemed to be largely affected by the notions of competition and team driving motivation to exercise, and therefore these should be considered when designing future programmes. As well as the positive influence of the Motivational aspects of SCC, Capability and Opportunity were also impactful, but these were also labelled as being prone to uncontrollable barriers to activity. Flexible workplace physical activity programmes like SCC have the potential to be effective at changing behaviours and increasing physical activity levels.

**Supplementary Information:**

The online version contains supplementary material available at 10.1186/s12889-025-25007-x.

## Introduction

Around 27.5% of adults worldwide are insufficiently active [1] and this figure increases to 37% in high-income countries. The benefits of physical activity and risks associated with sedentary behaviours on physical, mental, and cognitive health are well-known [2–5], which led the World Health Organization to recommend adults to do a minimum of 150 min of moderate intensity aerobic physical activity throughout week to achieve these benefits [5].

One potential factor impacting the prevalence of inactivity is that adults spend an average of 60% of their waking hours in the workplace [6], with many jobs being predominantly desk-based, particularly in many Western workplaces [7]. It has been estimated that in the UK, 84% of working hours are spent sedentarily [8]. Sedentary working conditions were further exacerbated when working from home [9–11] – which is particularly concerning given that working from home represented 40% of the UK workplace practices at the end of 2024 [12].

This high level of sedentary behaviour is a cause for concern within the workplace environment, making this a context of particular interest, and potentially contributing to the rise in the popularity of workplace physical activity interventions [13]. The effectiveness of these interventions at increasing physical activity levels varies, with each intervention having several components, including activity, intensity, duration and frequency [14, 15]. Additionally, with a number of different ways to measure activity levels, there is an extra level of potential confound [16]. For instance, a systematic review into an exercise programme for working women highlighted increased metabolic equivalence (METs), but no significant increase in minutes per week of moderate-vigorous intensity activity [17].

It has been highlighted that the easiest way for adults to engage in moderate intensity activity is by increased walking [18, 19]. Policymakers and stakeholders have called for increased numbers of interventions to incorporate walking, labelling it a ‘Best Buy for Public and Planetary Health’ [20]. Research has suggested that an added 75 min of brisk walking each week may be enough activity to elicit an extra 1.8 years of life – with 450 min garnering a further 4.5 years [21]. Not only is walking beneficial to health, but it provides an accessible way for most people to achieve activity targets, without requiring a high level of fitness, resource or free-time [22]. Additionally, a meta-analysis found that interventions that primarily promote walking rather than other forms of activity are significantly more effective [23] and they require little to no instruction to participants, specifically advocating for future support for walking or step count workplace interventions, and their fitness benefits.

One such workplace physical activity programme is the Step Count Challenge (SCC) which runs nationally across Scotland twice a year organised by third-sector organisation Paths for All (now called ‘Walking Scotland’ following rebranding; https://www.stepcount.org.uk/), frequently attracting participation from over 4000 employees nationwide. The challenge requires a self-determined team of five colleagues to complete the highest amount of physical activity possible, for either 8-weeks in Spring or 4-weeks in Autumn in competition with other teams across Scotland. The online system shows each participant their personal progression and how they are faring in comparison to their teammates, as well as how their team cumulatively compare amongst their workplace and nationwide against other teams. These leaderboards use step counts as homogenous units, however, participants can also record running, cycling, swimming and yoga, which is automatically converted to a walking step count equivalent. Participants can also convert additional activities to steps based on their metabolic equivalents, providing examples such as gardening, aerobic exercise classes and dancing. Activity can be added manually on the website or via linking wearable technologies for automatic synchronisation. Based on these reported activity levels, each week a daily step goal is set for each individual which can be amended by the individual to suit their goals. Additionally, on two occasions each week participants receive update emails from Paths for All that feature mini-competitions and tips for how to be active, as well as signposting to other helpful resources (i.e., blog posts and podcasts). The flexible nature of the challenge means that each workplace may promote and adopt the Step Count Challenge differently, with varying levels of engagement and collaboration from employers and within teams.

A review of behaviour change techniques used in workplaces interventions highlighted that the three most commonly incorporated behaviour change techniques in effective interventions are ‘Goal-setting’, ‘Providing instruction of how to perform a behaviour’ and ‘Prompting self-monitoring of the behaviour’ [24]. A further review added to this, finding that self-monitoring of the behaviour through the use of a wearable pedometer, in conjunction with goal-setting, are present in the most consistently effective interventions in terms of improving cardiorespiratory fitness [25]. This combination has frequently been found effective in improving a variety of outcome measures, including physical activity levels [26, 27]. Not only are these techniques present, but they essentially form the basis of the Step Count Challenge as a whole.

Our previous work showed that SCC is associated with increased physical fitness and mental wellbeing at a large-scale national level, with a further significant difference seen in the physical fitness levels of those who participated in a bespoke version of the SCC, compared to non-participants [28]. As part of the project, a qualitative study was also designed to explore the experiences of the participants in more detail using a theory-driven approach. Previous explorative qualitative interviews investigated the patient-reported health benefits of SCC participation, noting the abundant positive responses for areas of both physical and mental health impacts [29]. Further understanding these participant experiences can help to gain potential insight as to whether the SCC benefits them, especially when behaviour change theory is applied. For instance, the COM-B Model of Behaviour Change [30] states that no behaviour will happen unless the individual believes that they have the capability to perform it, the opportunity to do so, and feels motivated to carry it out. These 3 aspects represent the ‘C’, ‘O’, and ‘M’, which when present in synchronicity increase the likelihood of the B (Behaviour) occurring. Each of the ‘COM’ components can additionally be broken down further, acknowledging the vast array of potential influences on behaviour; Capability: Physical and Psychological; Opportunity: Social and Physical; Motivation: Automatic and Reflective. The application of the COM-B Model in a physical activity context has previously been shown to be effective and implementable in research by Willmott et al., who examined the model’s use against other behavioural theory as a framework for assessing both activity and eating behaviours [31].

In the present context, in terms of Capability, the SCC is centred around walking and wheeling, which have been acknowledged by researchers and policy makers as the most accessible forms of physical activity [19, 20]. Given the persistence of findings in the literature that so many people attribute their physical inactivity to a lack of time [32–35], it is clear that the ‘Opportunity’ element of the model is of critical importance, especially when put in the context of workplaces, where individuals often sit at a desk for around half of their waking hours. Therefore, when workplaces sign up to participate in the challenge, the social norms and culture are potentially altered, the social opportunities for physical activity are increased, and in some workplaces new physical opportunities are created within the working day, in an attempt to maximise a team/workplace’s chances at doing well in the competition. Additionally, given the unstructured nature of the SCC, which does not have set times for walking, or set activities that must be achieved on certain days or intensities, it provides participants with flexibility to complete the physical activities that they actually want to do, at a time that suits them. By centring the challenge around walking, this potentially makes it easier for participants to incorporate activity into their daily lives, rather than having to commit time out of their days to attend a specific exercise class. Finally, the idea of competition is likely to stimulate many individuals’ automatic motivations, due to humans’ innate desire to win and perform well. With each participant being able to see their position within their own team, their team’s position within the wider-workplace and the team’s national leaderboard position, some may consider there to be three competitions going on at once, depending on the mindset of the individual. Therefore, within the context, COM-B model has relevance to understand SCC further but has not been previously applied.

The aim of this study was to explore participants’ experiences of the SCC, and how participation affected their physical activity behaviours in comparison to pre-challenge habits guided by the COM-B model. However, given that the Challenge may have had lasting impacts and physical activity could take place outside the work hours or workplace, influencing their daily routine and lifestyle, we were also keen to find out about their general activity habits. The specific research questions were:

1. Does participation in the SCC impact physical activity behaviour?
2. What factors help or hinder physical activity during SCC participation?
3. What are the main motivations for physical activity and for participation in the SCC?

## Materials & methods

### Design & analysis

This qualitative study utilised semi-structured interviews with past participants of the SCC. Guided by the COM-B Model [30], we addressed elements of capability, opportunity, motivation, and behaviour with regard to physical activity and walking, and designed our questions in the context of the SCC (Supplementary File 1). While SCC is largely promoted as a workplace challenge, it clearly also impacts activity beyond the workplace, and so our questions addressed both work and non-work activity. Examples of how each element and sub-construct were addressed within the interviews can be seen in Table 1.

Transcripts were analysed using reflexive thematic analysis [36], which enabled searching for meaning within the data, examining explicit semantics, but also interpreting the more implicit, latent ideas. In line with Braun and Clarke’s [37] notion of a qualitative ‘Big Q’, the research was designed to focus on fewer participants, to enable generation of a deeper understanding of experiences and motivations of SCC participation. By adopting a reflexive thematic analysis approach, we accept an inherent subjectivity to our conclusions, however, we limited these through collaborative theme development (SW & GO) to achieve more nuanced understanding of the data.

**Table 1:** Examples of how each component and Micro-Construct of the COM-B model were addressed within the interview schedule

COM-B Element	COM-B Element Micro-Construct	Example Related Question
CAPABILITY	PSYCHOLOGICAL	• Are you aware of any national physical activity guidelines/recommendations, or any programmes other than the Step Count Challenge that target physical activity? • Do you tend to monitor your physical activity levels?
	PHYSICAL	• Is this type of physical activity something that you find comes naturally to you, that you feel comfortable and capable doing?
OPPORTUNITY	SOCIAL	• Did you incorporate walking into your daily life during the challenge (i.e. Active travel)? o Is this something that is easy to do, perhaps encouraged by your workplace, or something that you have to go out of your way to achieve?
	PHYSICAL	• Do you feel there are any barriers to physical activity? These can be physical or perceived o Are there any that effect you personally more than others? • Is there anything you wish you could do to increase your physical activity levels that isn’t already in place/accessible?
MOTIVATION	REFLECTIVE	• On the whole, what do you think the benefits of physical activity are to you? • How did you find the team element of the challenge?
	AUTOMATIC	• Do you feel as though you get rewarded for exercising? • Do you enjoy physical activity?
BEHAVIOUR		• How do you think that participating in the SCC impacted your physical activity levels during the event? o Do you think this was long-lasting impact, or mainly for the duration of the challenge?

We used a hybrid deductive-inductive analytic approach, first operating a bottom-up, inductive approach, based on both semantic and latent layers, without theoretical ties to COM-B model. We then additionally conducted a second level of analysis, coding with a top-down, deductive approach, looking solely for our theoretically-derived codes, which in this case were each element of the COM-B model (i.e., Social Opportunity). Using this hybrid approach, the process of coding needed to be organic and openly-iterative, with the six stages of thematic analysis proving to be a recursive procedure [38]. The reflexive nature of our approach enabled us to complete both levels of coding, before a repetitive process of grouping these into themes that best represented our data, corroborating and legitimating these themes to provide accurate representation of the true underlying meaning of the data. In terms of the theoretical perspectives adopted for this research study, given our desire to bolster our understanding of participant experience rather than describe a social reality [39], the work was conducted with a critical realist ontology, alongside an interpretivist epistemology. This allowed us to acknowledge the individual differences in everyone’s circumstances, and that each individual interprets reality differently, influenced by their own experiences and socio-cultural norms.

### Participants

15 participants (13 female, 2 male) were recruited between May - December 2022 having responded to advertisements shared via University staff newsletters, physical campus posters, and on Twitter/X (shared by the research team and Paths for All) with interviews taking place between July 2022 and January 2023. The inclusion criteria were being aged 18 and over and having previously participated in at least one SCC. One additional registered participant was omitted by mutual consent before participation due to an occupational conflict of interest (being a member of staff at Paths for All). The only demographic information formally collected for this study was gender, however, there was a range of ages and job types present in the sample (e.g. NHS staff, third-sector and council staff, university employees, etc.).

### Procedure

Participants took part in a one-to-one semi-structured interview with the researcher (SW). Fourteen participants opted for this via Microsoft Teams video call, with one choosing to be interviewed by phone call. Video calls are now frequently used for qualitative interviews, providing researchers the opportunity to talk face-to-face with participants when in-person methods are not possible [40]. This naturally suited the present study, as the SCC is a nationwide event, enabling participants from across Scotland to be interviewed despite the distance and COVID-19 safety concerns at the time of conceptualisation. One participant was unable to complete the interview on two occasions due to work emergencies, and so instead requested to be sent a digital version of the interview schedule and provided written responses. The interviews lasted between 19:34 and 43:17 (*M* = 28:07, *Mdn* = 27:07), and were recorded through both Microsoft Teams auto-recording (where relevant and possible) and Dictaphone, before being transcribed verbatim by SW.

Ethical approval for this study was obtained from the University’s School of Psychology & Neuroscience Ethics Committee (Approval Code: PS16058). All participants were informed that they had 7 days from the interview date to withdraw their data, after which all data was pseudonymised.

## Results

After conducting iterative hybrid deductive-inductive reflexive thematic analysis, we generated five salient themes to represent a coherent grounded interpretation of our data, which we then retrospectively named in accordance with elements of the COM-B model that we felt it most naturally represented, whilst not shoehorning the data. These themes were: (1) *Beliefs about capability impacted by experiences and performance*,* both personal and those of others*, (2) *Step Count Challenge presents the opportunity for a Physical Activity behaviour change*, (3) *General physical activity barriers*, (4) *SCC impacting Physical Activity Motivation*, and (5) *Why do we do it?*

### Theme 1: Beliefs about capability impacted by experiences and performance, both personal and those of others

This theme describes how a participant’s perceived capability may have been affected by their present personal circumstances, and the subsequent influence that this had on their activity behaviours. This reflects how not only is it our *actual* physical capabilities and knowledge that can help or hinder the likelihood of a behaviour, but also how this can be impacted by our psychological beliefs and confidence levels.

**Fig. 1:**
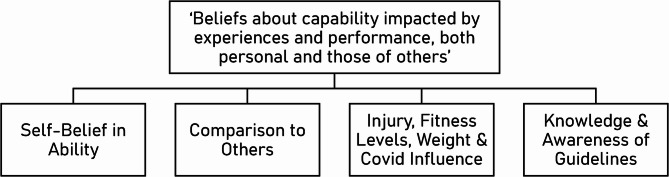
Thematic Map of Theme 1

Theme 1 was split into four sub-themes (See Fig. 1): Self-Belief in Ability; Comparison to Others; Injury, Fitness, Weight & COVID-19 Influence; and Knowledge and Awareness of Guidelines.

#### Self-Belief in ability

When discussing physical activity, it became clear that for the behaviour to happen, the individual needed to be confident that they had the ability to do it. Where self-belief, self-efficacy and confidence were not present, a participant’s motivation to do the subsequent activity dwindled and they were less likely to do it. ’Ability’ was largely focussed around having an adequate skillset to successfully complete the task (activity behaviour), and was apparent when discussing all physical activity, not just specific to the context of SCC.

*“I want to feel fit and healthy when I go and do stuff*. *Like*,* the more you do*,* the more you feel comfortable in doing it*,* sometime I go hill walking and you know… I can do it. And sometimes I’m with friends who are younger than me and who can’t do it … I’ve got a friend who’s 15 years younger than me and he is panting and out of breath when we go-… he hardly ever goes on the walks unless I sort of drag him. So like I never apologize for the walks*,* because I’m the older one*,* but I’m the fitter one*,* and I’m kind of glad about that”* – P14, M.

*“It was nice to go for longer walks and feel better and really understand*,* ‘Actually*,* no*,* you’re alright. You can do these things’”* – P13, F.

*“But walking’s the best thing*,* you know? It’s not a run*,* or it’s not a jog and you know*,* just like*,* anyone can walk”* – P15, F.

#### Comparison to others

This subtheme was SCC specific. It seems that the leaderboard aspect often led to individuals comparing themselves and their step counts with that of their team members. Although a motivating factor for some, the judging of step counts against that of others also discouraged others. This negative phenomenon was mostly reported by those who found themselves trailing in the team standings, which led to feelings of inadequacy and on some occasions resulted in participants looking to justify their comparatively low levels. Whilst many reported the comparison, whether this was used as motivation or deterrent for engagement was participant dependent.

*“I think the team that came first had done millions of steps in the first week and I thought ‘Ohh God*,* I can’t match that’ … You kind of tend to think ‘Well*,* I’m never gonna achieve that’. So*,* it’s just making the best of what I can do. You almost feel like giving up. Not in total*,* but just not actually pushing yourself quite as hard. You almost take a step back and think ‘ugh can’t be bothered’. If someone’s too far ahead of you*,* it’s a bit kind of demotivating”* – P2, F.

*“There’s some people that have got dogs here so they’re out walking their dogs every day and I dinnae have a dog*,* so like I cannae compete with that like. And we had some wee runners and we did it while they were running*,* that was the same. Ya kin? I’m a normal person me”* – P15, F.

#### Injury, fitness levels, weight, & COVID influence

Injury, fitness levels, weight, and COVID were also common points of discussion, where a participant explicitly mentioned how their physical activity behaviours were impacted by one or more of these factors. Ranging from physical injuries which prevented any physical activity, through to more psychological factors such as fears and doubts, references to barriers were again present when discussing activity habits. Interestingly, multiple participants did make a reference to the ‘catch-22’, whereby they were aware that the best way to tackle their issues was through physical activity, but these barriers were tough to overcome to get to that stage whereby improvements would be seen.

*“I broke my ankle in March*,* like smashed my ankle*,* I broke my tib and my fib in my heel and had some fairly major reconstructive surgery. So didn’t participate in the spring one*,* did the autumn one*,* you know sort of hobbling … Walking caused a lot of pain but I suppose psychologically*,* it made me do something*,* try something”* – P5, F.

*“I need to do more but you can’t do more because of your weight and what have you. So*,* the walking is-*,* it would be a brilliant thing if I did more of it because that would maybe bring my weight down and then following that you can do more. But at the moment I just*,* it’s about the only thing I can manage. I’ve got dodgy knees*,* so I mean I struggle to do any other sort of exercise apart from swimming*,* but I won’t put my swimming costume on so…”* – P8, F.

#### Knowledge & awareness of guidelines

This sub-theme is inherently linked with motivational elements, such that increased knowledge and awareness of physical activity guidelines and one’s own behaviours led to a tangible increase in motivation for several participants, as exemplified by the mediating relationship between Capability and Motivation within the COM-B model. It was noteworthy the ways in which participants reacted to awareness of their step counts with responses ranging from knowing their activity levels and the guidelines but not acting on it, all the way through to people who believed they were becoming almost obsessive about achieving their targets. This variation in response to the same knowledge further demonstrates how although knowledge and awareness are important, these do not automatically lead to a behaviour change, but may influence an individual’s motivation. There were also instances where during the SCC participants realised they had the means to monitor their activity levels, and used this as motivation during the challenge, but did not keep this up when there was no external pressure to do so.

*“It was very*,* very good because I had no idea how many steps I took in a day*,* because before the Step Count Challenge*,* I wasn’t so intensely aware of the need to walk. So*,* it was a very good awareness raising thing. … I assumed I got enough exercise*,* but actually on many a day you wouldn’t go anywhere near 10*,*000 on that … I would say I’ve become obsessive”* – P7, F.

*“After the challenge I carried on using my phone*,* still looking to see*-, *the steps had come down but I was still checking on what I was doing*,* seeing how far I was dropping*,* and now I can’t remember the last time I actually picked it up”* – P8, F.

### Theme 2: step count challenge presents the opportunity for a physical activity behaviour change

The notion of opportunity, or a lack thereof, is often seen as one of the biggest perceived barriers to physical activity [35, 41], impacted by both physical and social elements. When thinking about the physical aspects of opportunity, these refer primarily to one’s objective environment, whilst social opportunities denote the culture one finds themselves in, and the norms about the behaviour [30, 31].

**Fig. 2:**
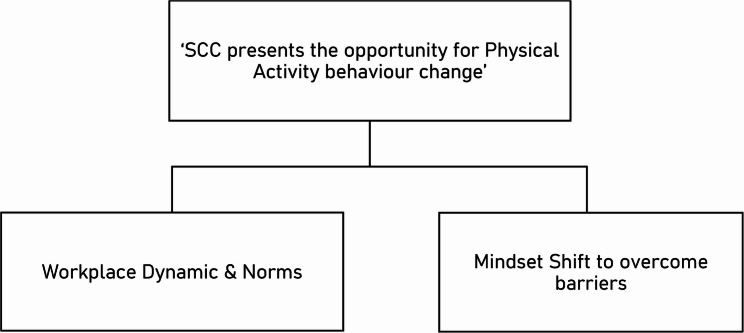
Thematic Map of Theme 2

Within the context of this study, the discussion around how the SCC provided an opportunity to change one’s usual habits or workplace culture was plentiful. The theme has been split into the two sub-themes (See Fig. 2), to capture the importance of individual mindset adjusted to see opportunity for physical activity where they perhaps did not previously, but also how a workplace participating was able to change the environment the employees found themselves in to be one far more conducive to physical activity.

#### Mindset shift to overcome barriers

When participating in SCC, many participants began to overcome obstacles that previously prevented physical activity. In this sense, SCC provided participants with an opportunity to look at their physical opportunities with a different lens. However, its impact was at times limited to the SCC duration.

*“I’m a fair-weather gardener*,* you know. But when the Step Count Challenge is on*,* you go out. You go out. Whether it’s wind or rain or hail or you know*,* you dress for the weather*,* you take that whole Billy Connolly attitude: There’s no such thing as bad weather*,* only bad clothing*,* you know? It forces you out… And you feel great afterwards”* – P5, F.

*“Just like finding the time*,* you know … Like the challenge is finished*,* so I haven’t been doing as much steps I did when I was doing it. Because it’s not there and it’s one of those like to get motivated”* – P3, F.

#### The impact and evolution of workplace dynamic & norms

This subtheme encapsulates where behavioural norms or standard practices in the workplace changed, both in terms of physical activity habits and social elements where the workplace dynamic, practices, and norms changed due to SCC participation. It was particularly noteworthy that there were differences noted between those participants reintegrated into a physical workplace having worked from home, and those where there was one team from a workplace, and the impact that this had on their physical and social opportunity to engage in physical activity.

*“Our manager*,* he encourages us on like our lunch*,* to go and get out the office. Like go walk*,* have a look round. We’re quite near [LOCATION] which is a really nice park in summer as well*,* it’s really nice. … if we have any events on at work*,* you know*,* he’ll book somewhere that’s like the other side of the park. So we have to walk like a 20 minute walk through*,* you know*,* but he’s quite good at encouraging active breaks as well”* – P11, F.

*“We did the group stuff at lunchtime*,* and then in the evening that’s when the mini challenge* [within the Team of 5] *kinda really stepped up. During the pandemic*,* so much of our working time that meant that we weren’t even walking from one room into the next to have a meeting*,* you know*,* walking to sit down again*,* everything was done on Teams*,* so you would sit on your computer for one meeting after another meeting after another meeting and… Just didn’t even have that ability just… to move to another room to sit back down again”* – P6, F.

*“Maybe because we only took part in one team. Maybe it was that it would be a different experience if it was like… more colleagues were taking part then*,* then maybe would’ve been more interactions. So*,* obviously we didn’t obviously know any of the other teams and things like that*,* so*,* you know*,* maybe it’s a different experience when*,* where*,* if we*,* you know*,* if we had joined and we had more than one team as an organisation there’s some more sort of banter or there’s more internal competition or something*,* maybe that makes it more interesting”* – P3, F.

### Theme 3: General physical activity barriers

**Fig. 3:**
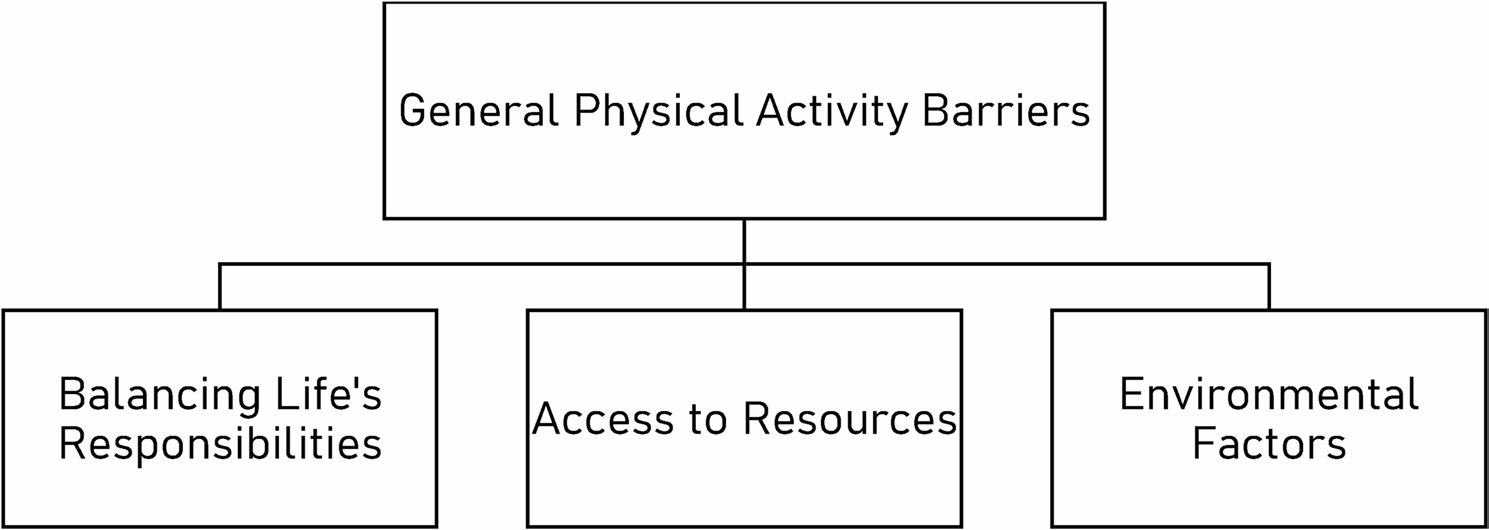
Thematic Map of Theme 3

The element of Opportunity was a popular discussion point throughout the interviews. Whilst SCC specifically warranted its own theme around barriers given the context, there was an additional need to acknowledge general barriers. Theme 3 covers those non-SCC specific barriers, that irrespectively occur all-year round. In this case, these included the impact of life responsibilities making physical activity a low priority task. Additionally, as expected, weather, safety, and access were also common responses (See Fig. 3).

#### Balancing responsibilities in the context of life

Many participants mentioned how low physical activity levels were attributed to having responsibilities of a higher priority. The participants reported lack of time for physical activity, due to work schedules and family commitments. However, there were also several participants who alluded to these barriers, but made it clear that overcoming these was achievable.

*“I think office environments aren’t really conducive to physical activity”* [In reference to the expectations and responsibilities of the job] – P5, F.

*“It’s quite heavy workload and restrictions*,* time scales and pressure as well. So sometimes you have to be logged on for like eight o’clock just to try and get through the day. And then obviously me and my wife*,* we’ll juggle dropping off the kids*,* picking them up after school*,* clubs*,* activities. So yeah*,* there is barriers*,* but I’ve found now I can still set myself aside an hour to do an activity if I can … We’ve always got that need to take the dog out”* – P10, M.

#### Access to resources

This subtheme relates to people’s accessibility to physical activity resources. Participants referred to this in positive and negative ways, with some reflecting why it poses a barrier to physical activity and others acknowledging their ease of accessibility and acknowledging that this was a privilege that not everyone had.

*“For me to go climbing or go swimming or go play badminton or anything it’s quite a trek*,* so not having any facilities nearby is a bit of a barrier to me doing slightly more organised exercise”* – P1, F.

*“I live in the countryside so that’s handy. You can just go for a walk … I’ve probably got about three*,* maybe four different places I could go first thing in the morning from where I am”* – P6, F.

#### Environmental factors

The environmental barriers to physical activity included poor weather conditions, darkness, and seasonal variation. It was important to capture these as the SCC occurs at two very different times of the year, with very differing environmental conditions. This distinction is especially the case in Scotland where daylight hours can vary considerably: for much of the Spring Challenge one could expect over 17 h of daylight, whereas by the end of the Autumn challenge, there are less than 8 h of daylight anticipated [42]. The lack of daylight can be a particular issue given that around 70% of SCC participants are female, and as reported by our participants, this can elicit safety concerns. Almost every participant commented on the impact that environmental factors had on their activity habits.

*“I would say obviously in Scotland*,* the weather is the biggest thing because you could have all the will in the world to want to go out for you know*,* a big hour long walk. But if it’s absolutely lashing down*,* it’s probably the last thing you want to do. I would say that I would say the weather was a bigger barrier than work”* – P9, F.

*“The spring one when days are longer*,* you tend to be out more anyways because the weather is better and just*,* you know*,* we tend to go for longer walks on the weekend. Whereas in October*,* it’s not really fun. In the wintertime it just feels like it’s constantly nighttime*,* which is quite depressing”* – P12, F.

*“Darkness is a wee bit different*,* you know for a woman*,* it’s a wee bit scarier when it’s getting dark”* - P15, F.

### Theme 4: Step Sount Challenge impacts motivation to be physically active

**Fig. 4:**
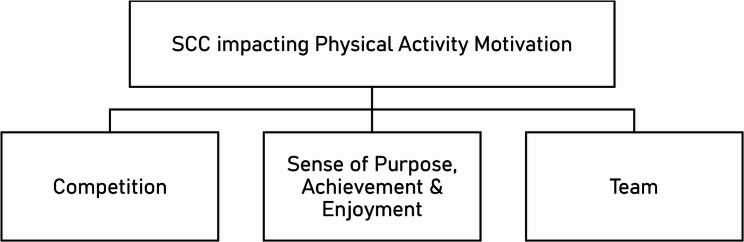
Thematic Map of Theme 4

The impact of SCC on motivation to be physically active was reflective of both intrinsic and extrinsic motivation, including elements of enjoyment and self-improvement, as well as the outcomes and external factors associated with completing the challenge. As shown in Fig. 4, there were three sub-themes derived here, corresponding to the influences of Competition, Sense of Purpose, Achievement and Enjoyment, and finally Team.

#### Competition

As SCC is a competition, it is unsurprising that this impacted motivation. This appeared to be a multi-level dynamic, whereby responses showed differing competitions of interest. Some only wanted the internal competition, for their own improvement and achieving their personal goals, thus operating on an intrinsic motivation level. However, many viewed their team of five as its own mini-competition, adding an extrinsic layer. There was also a desire to make sure that one’s team was the workplace winner - an element evidently lacking for those whose workplace only entered one team, and it became apparent that this impacted motivation. Interestingly, the nationwide leaderboard garnered little interest, with participants citing the lack of personal connection as the reason. Also noteworthy is the negative impact that some participants felt the competition had, reporting the obsession it instils, leading to them constantly checking the leaderboard to see if they were beating their perceived ‘opponents’.

*“I would say I’m quite a competitive person*,* but actually for this one*,* I was more focused on my own goals*,* you know*,* if I was sitting like maybe second or third*,* I was okay. I was okay with not being first. I was kind of more focused on my own goals. A couple of people got really into it*,* and they were like going out*,* doing like 15*,*000 steps every day”* – P9, F.

*“The competition between members in the team [was most important]*,* I didn’t really care about the other teams particularly. It was more our own team members*,* so you keep an eye on it. The first thing I did when I come in was like*,* look at it*,* and go ‘ahhhh ((gasp)) how did they manage to do that? Oh my God. I need to do more’. So it was quite stressful*,* but in a positive way*,* I suppose”* – P2, F.

“*There’s inevitably some degree of competition within your team*,* as well as against the other teams. The problem is with the other teams*,* you don’t necessarily really know who they are. So*,* you know*,* if they’re just from some random organization that’s not really connected to you … you might see the leaderboard*,* you’re number 132 or whatever it is but you don’t really know who these other people are”* – P14, M.

#### Sense of purpose, achievement & enjoyment

It was clear that for some participants, SCC gave them a reason to be physically active, providing a sense of achievement, and the feeling of being part of something bigger. The enjoyment that people got from their participation, and the positive reinforcement that came from it was clearly rewarding for some participants. This seemed to be motivation to continue and to increase their activity levels throughout the challenge.

*“I really liked having it graphed up being able to put in ‘today I did nine-thousand steps’ and being able to see my little number go up and see the fact that I was doing well and making my team do well” –* P1, F.

*“I think I probably hadn’t appreciated how huge it was until then*,* when it was actually on the website*,* and seeing how many teams there were*,* it was like ‘ohh*,* we’re a teeny*,* tiny little cog in there’ … Then there was just a whole n’other dimension and I’m thinking*,* ‘oh*,* I’m a part of something even bigger… that’s really good’” – P6*,* F*.

*“It was brilliant*,* I thoroughly enjoyed it… I mean we’ve still kept the good habit of going out every lunchtime”* – P2, F.

#### Team

The Team aspect had a pivotal role on participant motivation. Although there was motivation from trying to beat teammates as already discussed, many noted how being part of a team provided accountability and responsibility, or perhaps a social pressure to perform. Aside from this, participants reported how the team camaraderie and support positively motivated them to increase their activity levels. However, not every team experience was positive, with frustrations evident where it was perceived a teammate did not equally commit to the challenge.

*“I do like the team aspect… Because I think if you do it alone it wouldn’t have the same impact. You know*,* with a bit of friendly banter and friendly competition”* – *P12*,* F*.

*“It was a great experience to complete this challenge with colleagues. We all worked together*,* encouraging each other and congratulating each individual on their achievement … because I didn’t want to let my team down*,* I wanted to do my best for everyone” – P4*,* F*.

*“It just irritated me that they wouldn’t get their steps in … I just thought ‘come on*,* I can’t double my step count just to compensate for you guys who can’t be bothered to walk’”* – *P7*, *F*.

### Theme 5: Why do we do it?

**Fig. 5:**
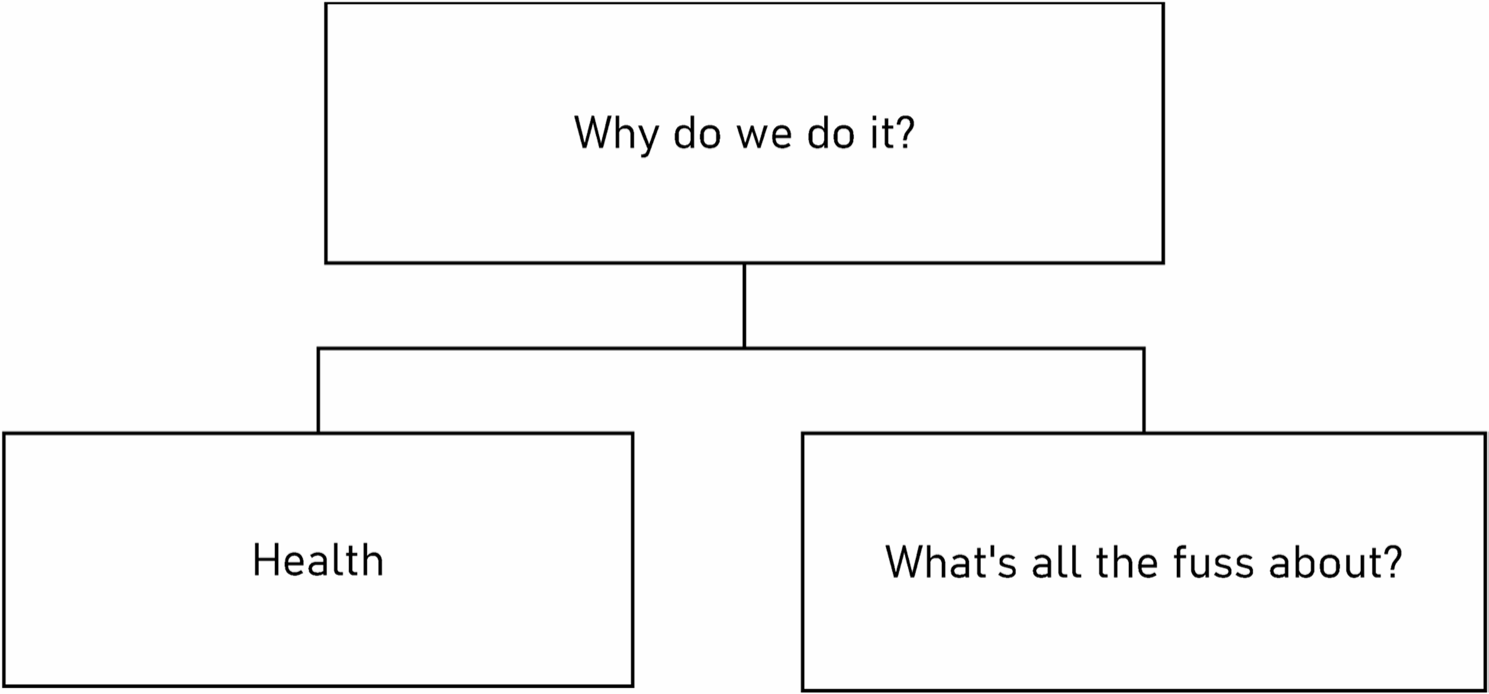
Thematic Map of Theme 5

As depicted in Fig. 5 below, Theme 5 represents the reasons for participation, including elements of health or curiosity. The former were largely extrinsic factors, such that the motivation was the perceived reward of health benefits, whereas the latter were those intrinsic motivations such as previous enjoyment and interest.

#### Health

A common response when asked about their main motivations for doing physical activity was the mention of expected health benefits, both physical and mental. In terms of physical health, responses covered motivation for activity to achieve weight loss, through to injury recovery and disease prevention. It was clear that everyone was aware of the positive health benefits of physical activity, and for many this was why they participate. In terms of mental health, many participants reported feeling happier after physical activity, and how they used it to relax or improve their wellbeing. Additionally, there were numerous references to the feelings of being present, more focused and engaged with one’s environment, giving participants the chance to get away from screens and into nature and green spaces.

*“In general*,* it’s more of a health thing*,* just trying not to turn into a sack of lard and getting heart disease and dying at fifty”* – P1, F.

*“You don’t wanna be a burden on health service. Obviously there’ll be times when you will be*,* but if you can avoid that*,* why not? You know*,* with something as simple as going out for a walk…”* – P6, F.

*“It’s good to like*,* connect with the outside world and see what’s going on*,* be more engaged with the surroundings and the people near me … Just detach myself from work*,* detach myself from my phone”* – P11, F.

#### What’s all the fuss about?

This subtheme was SCC specific, encapsulating where participants had seen others in the workplace do it before, or heard about other workplaces participating, and wanted to have a go themselves. There were also some participants whose workplaces had not participated before, and so their main reason for participating was because they were doing a pilot run for their organisations - wishing to understand the SCC, and its possible impact, investigating whether it was worthwhile implementing in their workplaces.

*“I know they’ve done it before and I didn’t do it the last time*,* so I just thought*,* “yeah*,* I’ll give it a go this time” and I’m really pleased I did. I knew that before when they’ve done it*,* there’d been a little bit of chat and encouragement of different teams*,* and I just wasn’t part of it. I think it definitely probably gave me the nudge to sign up”* – P6, F.

*“I think the charity that I work for*,* [WORKPLACE NAME]*,* they’ve previously done it*,* or some of the staff members have done it*,* but we’ve never done it as like a sort of organisation. So it was my idea*,* to kinda do it for fun*,* in the office”* – P11, F.

## Discussion

In order to holistically understand the impact that SCC participation has on participants’ wellbeing and physical activity, it is important to explore their experiences, in conjunction with health-based outcome measures [28]. By conducting interviews, we obtained rich data and novel insights into the influence that the SCC has on participant physical activity behaviour, generating five salient themes from analysis, each subsequently mapped to the COM-B Model for additional theoretical underpinning. This enabled us to theorise a potential understanding of how behaviour is affected by participation, with regards to elements of capability, opportunity, and motivation.

The study had three aims, the first of which was whether participation in the SCC impacted physical activity behaviour. It was evident from the responses of the participants that this was the case, and that their physical activity behaviour was positively affected, such that they were being physically active and reporting that SCC was either the cause or facilitator for this. It was also apparent that when discussing their physical activity behaviour that this was not limited to the workplace setting, suggesting that participation had an impact on physical activity in a more general way.

The second of the study’s aims was to identify the factors that helped and/or hindered activity during the SCC period, which was clear and evident throughout the findings. At each point throughout analysis, consideration was given to whether the factor being discussed was a facilitator, a barrier, or whether it came down to the individual’s own experience and circumstances, and both sides of each argument have been reflected throughout the results of this study.

Finally, the study aimed to gain an understanding as to the main motivations of participants, both in terms of the SCC participation, and also general physical activity. This was most demonstrably evident across Themes 4 and 5. With respect to the COM-B Model, it appeared that Motivation was actually the element most impacted by participation, with competition and team aspects proving particularly prominent rationales.

### Capability

The nature of the COM-B Model suggests that without the presence of the perceptions of Capability, a behaviour is unlikely to occur. Within this study, Capability was reflected by Theme 1, which encapsulated the popular discussion surrounding individuals’ belief in their ability; comparison to others; their physical ability; and their knowledge and awareness of the guidelines, and themselves.

Our ‘Beliefs about Capability’ theme not only captured the direct influence that Capability has on behaviour, but also through its interplay with motivation. Theme 1 unequivocally demonstrates the direct route of capability to behaviour, through its subtheme ‘Injury, Fitness Levels, Weight & COVID Influence’, in which responses were quite clear (i.e., *“I need to do more but you can’t do more because of your weight”* [P8]). However, encompassed within the sub-themes ‘*Self-belief in Ability’*, *‘Knowledge and Awareness’* and *‘Comparison to Others’*, the relationship between capability and motivation was also evident. For instance, in ‘Knowledge and Awareness’, it became apparent that some assumed they did enough activity, but until monitoring it for the SCC made them realise otherwise, they had not had the incentive to do more. This supports previous work exploring the SCC, which also highlighted that participants were attributing their participation in the challenge with helping them to realise their capabilities [29]. Furthermore, in terms of one’s self-belief in their ability, this was not confined to SCC periods. Discussing general habits, it was apparent that a lack of self-confidence and self-esteem limited motivation and prevented physical activity engagement. Alternatively, when discussing SCC specifically, the accessibility of walking enabled participants to successfully push themselves to do more than they thought they could, or normally would, subsequently enhancing self-belief, leading to greater motivation.

Arguably the most prevalent of Theme 1’s subthemes was ‘*Comparison to Others’*, though each participant reacted differently to these capability comparisons. For example, some participants acknowledged that others do more than them, believing these step counts were unattainable, and therefore did not try to, instead accepting feelings of an inability to compete. Conversely, several participants shared how this actually provided motivation - and providing further support for the bidirectional interrelationships of the COM-B Model aspects. In this context, participation in the challenge sometimes provided the motivation to try a behaviour, which then enhanced sense of capability.

### Opportunity

Two themes were attributed to Opportunity – ‘Step Count Challenge presents the opportunity for physical activity behaviour change’ and ‘General Physical Activity Barriers’. Generally speaking, our sample’s responses reflected existing literature; highlighting a lack of time [35, 43], workload [6], weather [44], darkness-related safety concerns [45], resource issues [46]and family responsibilities [47]. Suitably for the present context, participants were frequently highlighting work-related issues as prominent barriers to physical activity – precisely what the SCC targets.

When qualitatively investigating workplace physical activity interventions, Ojo et al. concluded that workplace environments need to become more conducive to activity, such that employees are afforded more opportunities to be less sedentary [48]. They further identified the desire from their sample for the social opportunity to facilitate increased activity on a team basis. The importance of this is that the present study identified a similar notion, in the context of a real-world, team-based programme, as Ojo et al.’s participants wished for. It is therefore interesting to see how their suggestions for improving workplace physical activity work in practice. The subtheme *‘SCC presents the opportunity for physical activity behaviour change’* highlighted how there was a positive environment shift in the workplaces, whether that be increasingly walking around the office whenever possible; lunchtime group walks; or getting pressure/permission from management. However, the workplace environment did not change for everyone. Remote working impacted the SCC’s ability to create an active-minded work environment, perhaps through the absence of immediate external pressures. Additionally, where a workplace only entered one team, there was a paucity of support from their social environment, and participation was perceived to be less engaging.

Workload, time, and workplace environment were also previously identified as being barriers to the reduction of workplace sitting [49]. However, the flexible nature of SCC may enhance its chances of success, given that participants can be active at a time that suits them. Therefore, they have an opportunity to exercise irrespective of their workload, as well as having a potentially more supportive workplace environment.

Of course, environmental factors (such as weather) were also greatly important, and of particular importance in the context of Scotland. In fact, only one participant did not mention environmental factors as being detrimental to exercise habits. Besides the weather, safety concerns around darkness were also mentioned by multiple participants. To contextualise this, Scotland’s northern latitude makes its climate particularly prone to extremes in terms of temperature, daylight, and precipitation, varying significantly across the year, providing many potential barriers to exercise [50]. This is especially important to consider given that for much of the Spring Challenge one could expect over 17 h of daylight each day, whereas by the end of the Autumn challenge, there are less than 8 h of daylight expected [42], which are likely to coincide with the working hours of many.

Whilst Scotland’s climate brings potential barriers, many participants were also commenting on the impact that its landscape has on their access to resources. For instance, participants mentioned how living in the countryside or near water provided opportunity to access free resources for exercise. However, those in urban areas noted the difficulty accessing resources, and that even those manufactured resources (e.g., parks and gyms) required extra time and effort, therefore having a negative influence on the individuals’ motivation. One participant acknowledged the idea that despite having access to a local gym, going from artificial light in the office to more artificial light in the gym in the evening was not desirable. Whilst this is necessary within winter months in Scotland to provide adequate brightness for both leisure and work activities, it clearly is not preferential, and the benefits of exposure to natural light are known to be profound [51, 52].

Alongside the workplace dynamic, many participants also commented on their own personal mindset shifts, and how participation in the challenge led them to overcome barriers that might normally prevent them exercising. The most obvious account of this was provided by Participant 5 who was very definitive in their mindset shift - “when the Step Count Challenge is on, you go out. It forces you out, and you feel great afterwards”. This was possibly the most direct, but responses of this nature were common, noting how they either grin-and-bear the Scottish weather, or plan ahead for when the weather is forecasted to be the ‘nicest’. Of course, the weather was not the only example of a barrier that participants were finding ways to overcome because of SCC. For instance, some participants decided to do work meetings outside as walking meetings, and others were opting to take work-based video calls on exercise equipment.

Collectively, these responses are extremely positive and supportive of SCC, providing a strong sense that SCC presented the opportunity for increased physical activity levels.

### Motivation

Motivation is crucial within the COM-B Model, offering a direct and mediating route to behaviour. Within this study, Motivation was the most prominent discussion point. Whilst not every participant expressed the same motivation, everyone had a reason to participate, and without that it was unlikely for any amount of Capability or Opportunity to make the final behaviour occur.

Willmott et al., found Motivation had a significantly positive relationship with physical activity behaviour levels [31]. Interestingly, the individual indicators of motivation that they found to be significantly related included ‘Self-Efficacy’, ‘Identity’, ‘Positive Affect’, and ‘Intentions’, which can also correspond to our own themes and subthemes. The inclusion of self-efficacy here is another demonstration of the link determined between Capability and Motivation, such that those with the belief that they can adequately perform a behaviour are also more likely to want to do it.

Identity was present within the SCC primarily through the ‘*Team’* aspect, providing some participants with the motivation needed. It has been suggested that a sense of team identity can promote engagement with physical activity behaviour [53], possibly through introjected regulation, whereby an individual does a behaviour to avoid the feeling of guilt or shame [54]. However, this clearly was non-homogenous, as it was noted that some team members gave up or were not taking it seriously. This can subsequently impact on their teammates’ motivation. For instance, Participant 7 refused to maintain doing additional activity “just to compensate for you guys who can’t be bothered” in order to keep their team competitive. Regrettably they continued to explain that this perceived lack of commitment from their teammates to contribute to the competition led the participant to choose not to participate in future SCCs. However, the individual attributed SCC with providing long-term activity level awareness and motivation to exercise, despite their lack of future participation.

‘Positive Affect’ was also present within our data, contextualised across multiple subthemes, that is, ‘*Sense of Achievement*,* Purpose & Enjoyment’*, and ‘*Health’*. Whilst semantically many labelled their SCC experience ‘fun’, it was also clear that some were lacking this sense of enjoyment with general activity. Multiple participants discussed how although they feel better about themselves after exercising, they do not actively enjoy it, expressing their resultant lack of motivation to be a large barrier. In contrast, some participants found being outside in nature whilst exercising in green and blue spaces was enjoyable enough to do it frequently, which would also be beneficial for their mental health and productivity too [55].

Warranting a subtheme of its own, ‘*Competition’* is a large aspect of SCC, operating on a multi-level dynamic. At one level, several participants noted their primary motivation was self-improvement, and so their only competition was against themselves. Sedikides and Hepper [56] suggest that those who receive internal improvement-orientated feedback and are successful at self-improvement subsequently experience an increase in positive affect – which as previously stated, leads to further increased motivation. Although, this can also work in reverse, such that those who do not succeed to achieve their goals can also become less motivated.

Competition as a motivator was also seen more conventionally, whereby participants were trying to beat others, including colleagues, other teams in their workplace, and the other teams nationwide. The idea of competition as being motivational for physical activity is not novel [57], but is potentially underutilised in workplace programmes. Many programmes incorporate social support through group activity which can be beneficial for the encouragement of exercise behaviour changes [58], however the importance of social comparison and competition has frequently been reported [59, 60]. A previous study investigating the influence of social support and social comparison on activity levels found in conditions where participants had a competitive element, physical activity levels were significantly higher than a control group [61]. SCC can achieve both, through the supportiveness of the ‘Team’, social comparison, and competition.

However, forming the social community element may prove difficult to achieve in an online-based programme like SCC. Multiple participants referenced the lack of care about teams they did not know, suggesting a lack of community in this sense. Furthermore, and especially for those who were falling behind in their respective competitions, the comparison with others occasionally proved negative, possibly stemming from the perceived undesirable feeling of losing, in instances quitting seemed more desirable.

###  Strengths & limitations

This qualitative study is the first to explore the experiences of SCC participants, from a behaviour change theory perspective. The ontological and epistemological positions taken allowed the researchers to be open-minded about the participant responses and understand that we all have differing take-aways from our experiences, enabling both semantic and latent analyses, to deduce barriers and facilitators to gain a deeper understanding of the participants’ journey. Additionally, though being grounded in theory was a definite strength of the research, it was not the epitome of the analytical process, such that we were able to conduct a hybrid approach, with both inductive and deductive coding. The strength of these codes, and the derived themes, was further validated by the co-produced nature of them between multiple researchers, each of whom had their own personal experiences and biases.

We recruited a sample of 15 participants and whilst acknowledging everyone has their own experiences, we are confident that few new codes are likely to be created from the latter interviews. The repetitive recurrence of our key themes provides reassurance that our participants were representative.

It is possible that anyone willing to discuss their experience likely had positive ones, especially given that some mentioned how those who did not enjoy the challenge were prone to dropping out early, and were thus unlikely to acknowledge post-challenge advertisements. However, there were participants who were not stereotypically eager participants or particularly active, who willingly shared negative aspects of their SCC experience.

Additionally, other than gender, we did not collect any extra demographic information or information about their SCC performance, which may have been useful in providing us the opportunity to compare the responses of those who were particularly active or not, rather than deducing this from their responses.

##  Conclusions

This study aimed to explore participant’s experiences of SCC, and how participation affected physical activity habits. On the whole, participants reported that their physical activity levels improved during SCC. Seemingly the main reason for this was the added motivation given by the competitive element, which was supported by the team aspect. Whilst there were several Capability and Opportunity influences, Motivation having the greatest impact was apparent. However, looking at longer-term effects, these were not homogenous. Some highlighted a change of habits due to participation, and some shared that their motivation ended with the SCC - further evidence that human behaviour cannot be considered generalisable or homogenous, and nor should practitioners try to assume it is when creating programmes.

This study adds support to the previous literature around physical activity programmes, and shows that the participants themselves largely welcome competitive and team elements, finding this beneficial to their motivation. Addressing this in a workplace setting, using a flexible programme allowed participants the opportunity to make a change in physical activity behaviour that is not limited to the workplace itself. However, barriers remain regardless, many of which are uncontrollable (i.e., Scottish weather). Nevertheless, participant responses provided support for the implementation of a flexible physical activity programme, where environment and social norms become positively charged towards exercise. It is imperative that policymakers and employers consider the implementation of such programmes, enabling employees the opportunity to engage in physical activity, preferably with one’s colleagues.

## Supplementary Information

Supplementary Material 1.

## Data Availability

The transcript data for this study cannot be made available for sharing due to lack of given consent from participants for their data to be placed in a publicly accessible data repository.
