# Supplementary material for: “I think office environments aren’t really conducive to physical activity”: a qualitative interview study with participants of a workplace physical activity programme
Source: BMC Public Health. 2025 Nov 6;25:3803. doi: 10.1186/s12889-025-25007-x (PMC12590697; doi:10.1186/s12889-025-25007-x)
Supplement: Supplementary file 1 — Supplementary Material 1. [file 12889_2025_25007_MOESM1_ESM.docx]

**Intro/Rapport:**

**Background/Experience:**

WORK

- Desk based or active?
- FT or PT?

SCC EXPERIENCE

- How many times?
- Both Seasons?
- Enjoy it?
- Same team?
- Captain?
- Impact on PA? Long-lasting or just challenge duration?
- Physical Health impact?
- Mental Wellbeing?

MAIN METHOD OF EXERCISE

ROUGH STEPS PER WEEK ESTIMATE?

**Capability:**

COMFORTABLE/CAPABLE?

MONITOR DURING NON-SCC TIMES?

- Set targets etc?
- Change if not what you want?

CONSCIOUS INCLUSION IN DAY OR JUST ROUTINE?

AWARE OF GUIDELINES OR ANY OTHER PA ENHANCING PROGRAMES?

**Opportunity:**

INCORPORATE INTO DAILY LIFE DUIRNG CHALLENGE?

- ENCOURAGED BY WORKPLACE?

ROUTINE?

- REGULAR TIME OR DAY FOR EXERCISE?
  - Structured/organised events?
- SAME ROUTE/LOCATION?
  - Why?

INDIVIDUAL OR GROUP?

- Preference or Necessity?
- Benefits?

BARRIERS?

**Motivation:**

GROUP VS INDIVIDUAL MOTIVATION?

MAIN MOTIVATIONS FOR EXERCISING?

REWARD?

SAME EXERCISE EVERY TIME?

BENEFITS OF EXERCISE?

WHY DID YOU PARTICIPATE IN SCC?

TEAM EXPERIENCE?

- Mini Competition or Team event for joint goal?
- Benefit?
  - Hindrance?

**Final Thanks and Debrief**
